# Impact of Hepatitis C Virus Clearance on Cardiovascular Risk: A Real‐World Experience From the Nationwide Taiwan Hepatitis C Virus Registry

**DOI:** 10.1002/kjm2.70036

**Published:** 2025-05-24

**Authors:** Ping‐Jen Hu, Pei‐Chien Tsai, Chi‐Yi Chen, Hsing‐Tao Kuo, Chao‐Hung Hung, Kuo‐Chih Tseng, Hsueh‐Chou Lai, Cheng‐Yuan Peng, Jing‐Houng Wang, Jyh‐Jou Chen, Pei‐Lun Lee, Rong‐Nan Chien, Chi‐Chieh Yang, Gin‐Ho Lo, Jia‐Horng Kao, Chun‐Jen Liu, Chen‐Hua Liu, Sheng‐Lei Yan, Chun‐Yen Lin, Wei‐Wen Su, Cheng‐Hsin Chu, Chih‐Jen Chen, Shui‐Yi Tung, Chi‐Ming Tai, Chih‐Wen Lin, Ching‐Chu Lo, Pin‐Nan Cheng, Yen‐Cheng Chiu, Chia‐Chi Wang, Jin‐Shiung Cheng, Wei‐Lun Tsai, Han‐Chieh Lin, Yi‐Hsiang Huang, Ming‐Lun Yeh, Chung‐Feng Huang, Meng‐Hsuan Hsieh, Jee‐Fu Huang, Chia‐Yen Dai, Wan‐Long Chung, Yen‐Hsiange Wang, Ming‐Lung Yu, Ming‐Jong Bair, T‐COACH Study Group

**Affiliations:** ^1^ Department of Internal Medicine, Division of Gastroenterology Shuang Ho Hospital, Taipei Medical University New Taipei City Taiwan; ^2^ Hepatobiliary Section, Department of Internal Medicine, and Hepatitis Center Kaohsiung Medical University Hospital; Hepatitis Research Center, School of Medicine and Cohort Research Center, Kaohsiung Medical University Kaohsiung Taiwan; ^3^ Department of Internal Medicine Chiayi Christian Hospital Chiayi Taiwan; ^4^ Division of Hepatogastroenterology, Department of Internal Medicine Chi‐Mei Medical Center Tainan Taiwan; ^5^ Division of Hepatogastroenterology, Department of Internal Medicine Chiayi Chang Gung Memorial Hospital Chiayi Taiwan; ^6^ Department of Gastroenterology Dalin Tzu Chi Hospital, Buddhist Tzu Chi Medical Foundation Chiayi Taiwan; ^7^ Division of Hepatogastroenterology, Department of Internal Medicine China Medical University Hospital Taichung Taiwan; ^8^ Division of Hepatogastroenterology, Department of Internal Medicine Kaohsiung Chang Gung Memorial Hospital and Chang Gung University College of Medicine Kaohsiung Taiwan; ^9^ Division of Gastroenterology and Hepatology Chi‐Mei Medical Center Tainan Taiwan; ^10^ Division of Hepatology, Department of Gastroenterology and Hepatology Linkou Medical Center, Chang Gung Memorial Hospital Keelung Taiwan; ^11^ Division of Gastroenterology, Department of Internal Medicine Show Chwan Memorial Hospital Changhua Taiwan; ^12^ Division of Gastroenterology and Hepatology, Department of Medicine E‐Da Hospital, I‐Shou University Kaohsiung Taiwan; ^13^ Graduate Institute of Clinical Medicine, National Taiwan University College of Medicine Taipei Taiwan; ^14^ Division of Gastroenterology and Hepatology National Taiwan University Hospital Taipei Taiwan; ^15^ Division of Gastroenterology, Department of Internal Medicine Chang Bing Show‐Chwan Memorial Hospital Changhua Taiwan; ^16^ Division of Gastroenterology, Department of Internal Medicine Changhua Christian Hospital Changhua Taiwan; ^17^ Division of Gastroenterology, Department of Internal Medicine MacKay Memorial Hospital Taipei Taiwan; ^18^ Department of Internal Medicine St. Martin De Porres Hospital–Daya Chiayi Taiwan; ^19^ Division of Gastroenterology and Hepatology, Department of Internal Medicine National Cheng Kung University Hospital, College of Medicine, National Cheng Kung University Tainan Taiwan; ^20^ Division of Gastroenterology, Department of Internal Medicine Taipei Tzuchi Hospital, the Buddhist Tzuchi Medical Foundation New Taipei Taiwan; ^21^ Division of Gastroenterology and Hepatology, Department of Medicine Kaohsiung Veterans General Hospital Kaohsiung Taiwan; ^22^ Division of Gastroenterology and Hepatology, Department of Medicine Taipei Taiwan; ^23^ Institute of Clinical Medicine, National Yang‐Ming University Taipei Taiwan; ^24^ Health Management Center Kaohsiung Medical University Hospital Kaohsiung Taiwan; ^25^ School of Medicine, College of Medicine Taipei Medical University Taipei Taiwan; ^26^ Division of Gastroenterology, Department of Internal Medicine Taitung Mackay Memorial Hospital Taipei Taiwan

**Keywords:** cardiovascular disease, hepatitis C, interferon therapy, stroke, sustained virologic response

## Abstract

Hepatitis C virus (HCV) infection is associated with an increased risk of cardiovascular disease (CVD); however, the impact of interferon (IFN)‐based therapy on cardiovascular outcomes remains unclear. This nationwide cohort study included 7411 patients with HCV from The Taiwanese Chronic Hepatitis C Cohort registry who received IFN‐based therapy between 2003 and 2014. Patients were categorized into sustained virological response (SVR) (*n* = 5785) and non‐SVR (*n* = 1676) groups. The incidence of new‐onset CVD events, including stroke, coronary artery disease, heart failure, and arrhythmia, was assessed using three Cox proportional hazard models adjusted for different sets of confounding factors. The cumulative CVD incidence was comparable in the SVR and non‐SVR groups (11.2% vs. 10.2%, *p* = 0.609). SVR was not significantly associated with a reduced overall CVD risk among the three models [hazards ratio (HR) = 0.88, 95% confidence interval (CI): 0.71–1.05, *p* = 0.158]. However, a lower risk of stroke was observed in patients who achieved an SVR, although the difference was not significant (HR = 0.84, 95% CI: 0.74–0.94). The results of the sensitivity analyses confirmed these findings. An SVR following IFN‐based therapy did not substantially reduce the overall CVD risk; however, a potential reduction in stroke risk was observed. These results emphasize the importance of long‐term cardiovascular risk assessments and highlight the need for further research, particularly in the direct‐acting antiviral era in which increased cardiovascular benefits may be expected.

## 1 Introduction

Hepatitis C virus (HCV) is a major cause of chronic liver disease, globally affecting approximately 71 million individuals [1]. In addition to its progression to cirrhosis and related complications, HCV infection is linked to various extrahepatic manifestations, including insulin resistance [2], renal dysfunction [3], cryoglobulinemia [4], central nervous system abnormalities [5], and cardiovascular disease (CVD) [6]. The accumulating evidence suggests that HCV infection is an independent risk factor for CVD and is associated with increased cardiovascular‐related mortality [6].

A large‐scale study analyzing 82,083 and 89,582 people with and without HCV, respectively, demonstrated a 1.25‐fold increased risk of coronary artery disease in those with HCV, even after adjusting for the traditional cardiovascular risk factors [7]. Similarly, a Taiwanese cohort study reported that individuals with HCV had a nearly 1.5‐fold higher risk of developing peripheral artery disease (PAOD) than healthy controls, with the risk increasing with age [8]. The mechanisms underlying the elevated cardiovascular risk in people with HCV likely involve both direct and indirect metabolic pathways. These pathways include endothelial injury, chronic inflammation, increased insulin resistance, and direct vascular invasion by the virus [9]. Furthermore, these metabolic alterations, when compounded by lifestyle factors, contribute to the increased cardiovascular risk observed in this population.

HCV eradication with pegylated interferon (IFN)‐based therapy is associated with improvements in intrahepatic and extrahepatic outcomes. These improvements include a reduced risk of hepatocellular carcinoma, liver cirrhosis, end‐stage renal disease, diabetes, lymphoma, and all‐cause mortality [10, 11, 12]. Regarding cardiovascular outcomes, successful HCV treatment is associated with a lower risk of CVD. However, El‐Dosouky et al. [13] reported that IFN‐based therapy may be associated with various cardiac complications. A large retrospective cohort study found no significant reduction in CVD risk among patients who achieved an SVR compared with those who did not [14]. These findings highlight the ongoing uncertainty regarding the effects of antiviral therapies on cardiovascular outcomes.

Direct‐acting antivirals (DAAs) are currently the primary treatment for HCV infection, offering high SVR rates and fewer adverse effects than IFN‐based therapies. Additionally, the risk of HCV recurrence following DAA treatment is lower than that observed with IFN‐based regimens [15]. Historically, IFN was the sole available treatment for HCV eradication. However, IFN use is associated with numerous adverse effects, including fatigue, influenza‐like symptoms, hematological abnormalities, and neuropsychiatric complications [16]. Moreover, adverse cardiovascular effects are frequently observed in patients with chronic hepatitis C receiving IFN therapy [17]. These cardiovascular complications may persist even after achieving an SVR, potentially influencing their long‐term cardiovascular outcomes [13]. Consequently, whether IFN‐mediated HCV eradication effectively reduces CVD risk remains unclear and requires further investigation.

In this study, we aimed to evaluate the incidence of CVD events among people with chronic HCV infection in this large, real‐world cohort study by comparing those who achieved an SVR with those who did not. Subgroup analyses were conducted to assess the effects of successful antiviral therapy on new‐onset CVD development. Stratified analyses based on SVR status were performed to identify the independent risk factors associated with CVD events. We used comprehensive demographic, laboratory, and clinical data, including data related to the treatment response to IFN‐based therapy. All the data were retrieved from the nationwide multicenter cohort, “The Taiwanese Chronic Hepatitis C Cohort (T‐COACH)” and the “National Health Insurance Research Database (NHIRD).”

## 2 Methods

### 2.1 Study Cohort

Eligible patients were identified from the nationwide multicenter cohort T‐COACH (The Taiwanese Chronic Hepatitis C Cohort). This cohort enrolled 15,836 patients with HCV infection from 23 regional hospitals and medical centers across Taiwan between January 2003 and December 2014, representing approximately 21% of all treated HCV patients in Taiwan during this 12‐year period (http://data.nhi.gov.tw/).

**FIGURE 1:**
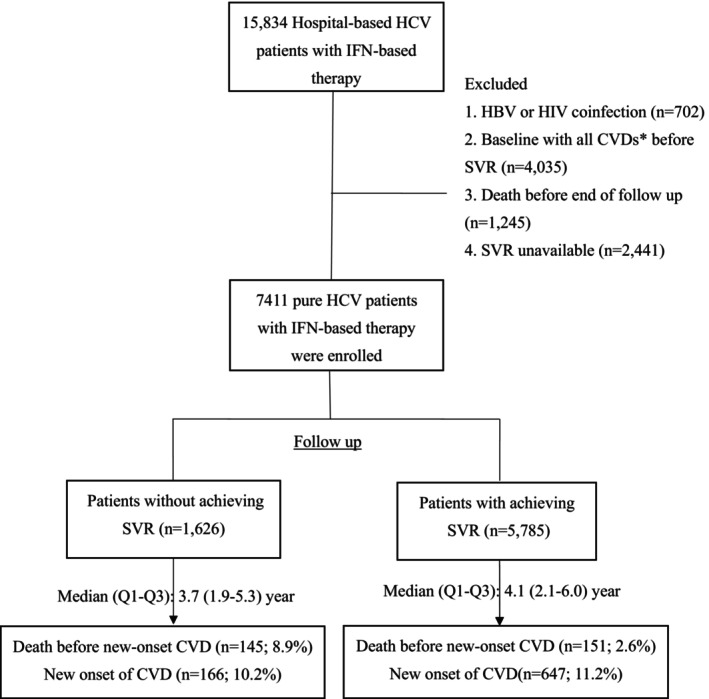
The flow chart of this study. All CVDs including stroke, peripheral arterial occlusive disease, heart failure, arrhythmia, cerebrovascular accident, and coronary artery disease. CVD, cardiovascular disease; HBV, hepatitis B virus; HCV, hepatitis C virus; HIV, human immunodeficiency virus; IFN, interferon; SVR, sustained virologic response.

The key eligible criteria for patients included in this study are as follows: (i) age > 20 years; (ii) patients with HCV diagnosed by liver histology or anti‐HCV seropositivity for > 6 months; (iii) seropositivity for HCV RNA; and (iv) patients who had received IFN‐based therapy for at least 4 weeks. Patients with human immunodeficiency virus or hepatitis B virus coinfection (*n* = 702), baseline with cardiovascular disease (defined as International Classification of Diseases, Ninth Revision (ICD‐9) codes 410–414, 426–428, 430–438, 440.2, 440.3, 440.8, 443.81, 443.89, 443.9, 444.22, 444.8, 447.8, and 447.9, *n* = 4035), death before the end of follow‐up period (*n* = 1245), and virologic outcomes unavailability (*n* = 2441) were excluded. Finally, a total of 7411 patients were enrolled in this further study (Figure 1).

For all patients, detailed host characteristics (including demographics, medical history, laboratory findings, comorbidities, and cirrhotic status) and virological parameters (including HCV genotype, viral load, and treatment response) were recorded before and after antiviral therapy. SVR was defined as undetectable HCV RNA 24 weeks after the completion of antiviral therapy.

This study was approved by the Institutional Review Board (IRB) of each participating hospital, in compliance with the International Conference on Harmonization guidelines for Good Clinical Practice. All patients provided written informed consent prior to study enrollment.

### 2.2 Linked to National Health Insurance Research Database and Study End Point

Taiwan's National Health Insurance (NHI) program, implemented in March 1995, provides universal healthcare coverage to over 99% of Taiwan's 23 million residents. The NHI system maintains a comprehensive research database, which includes demographic characteristics, diagnostic codes, surgical and medical procedures, prescriptions, laboratory test results, and death registry data. In this study, NHIRD data were linked until December 31, 2015. Disease diagnoses in this study were coded using the ICD‐9. Cardiovascular events included stroke, PAOD, heart failure, arrhythmia, cerebrovascular accident, and coronary artery disease. All diagnoses were confirmed based on at least three outpatient visits or one hospitalization (Table S1). The follow‐up duration was determined using ICD codes from the NHIRD, tracking patients from their enrollment in the T‐COACH cohort until death or December 31, 2015, whichever occurred first.

The primary study endpoint was the development of CVD events among patients with chronic HCV infection, comparing those who achieved SVR and those who did not follow IFN‐based therapy.

### 2.3 Basic Characters and Laboratory Data

Patient demographic characteristics, medical history, clinical features, and laboratory data were collected. The host profiles included age, sex, body mass index (BMI), complete blood count, biochemical markers, renal function, and liver cirrhosis status. Virological characteristics included HCV genotype and viral load. Medical history was assessed, including diabetes mellitus, hypertension, and hyperlipidemia. Statin and aspirin use were defined as at least one prescription within 180 days prior to the index date. BMI was calculated as weight/height^2^ (kg/m^2^). Estimated glomerular filtration rate (eGFR) calculated according to the Taiwanese Modification of Diet in Renal disease: eGFR = 186 × serum creatinine [mg/dl]−1.154 × age [years]−0.203 × 0.742 (if female) [18]. Fibrosis‐4 index (FIB‐4) was calculated as the formula: age (years) × aspartate aminotransferase [U/L]/platelets [10^9^/L] × (alanine aminotransferase [U/L]^1/2^) to evaluate hepatic fibrosis. Liver cirrhosis was defined as any of the following: liver histology [19], transient elastography (FibroScan; Echosens, Paris, France) > 12 kPa [20], acoustic radiation force impulse > 1.98 m/s [21], FIB‐4 > 6.5 [22], or the presence of clinical, radiological, endoscopic, or laboratory evidence of cirrhosis and/or portal hypertension.

### 2.4 Statistical Analysis

Categorical variables were expressed as number (percentage), while continuous variables were reported as mean ± standard deviation (SD). Differences between groups were assessed using Pearson's chi‐square (*χ* ^2^) test (or Fisher's exact test when expected cell count < 5) for categorical variables, and Student's *t*‐test (or analysis of variance (ANOVA)) for continuous variables. Person‐years were calculated from the start of antiviral therapy to the first occurrence of CVD events, death, or December 31, 2015, whichever came first. The annual incidence of CVD events was determined as the number of new‐onset cases divided by the total person‐years of follow‐up. Missing values for continuous variables were imputed using the mean value.

### 2.5 Survival Analysis and Risk Assessment

Death was considered a competing risk event; therefore, we employed Gray's cumulative incidence method to modify the Kaplan–Meier survival analysis [23]. The incidence of CVD events was compared between patients who achieved SVR and those who did not.

To estimate hazard ratios (HRs) with 95% confidence intervals (CIs), Cox proportional hazards models were applied, incorporating univariate and multivariate‐adjusted analyses [24]. Three Cox models were constructed:

- Model 1: Adjusted for age, sex, BMI, statin use (yes/no), and aspirin use (yes/no).
- Model 2: Further adjusted for FIB‐4, eGFR, and HCV genotype (1 vs. non‐1).
- Model 3: Additionally adjusted for diabetes mellitus (yes/no), hypertension (yes/no), and hyperlipidemia (yes/no).

Subgroup analyses were conducted to evaluate the impact of successful antiviral therapy on new‐onset CVD events among specific patient populations. To ensure the robustness of our findings, sensitivity analyses were performed by excluding CVD events occurring within the first year post‐antiviral therapy.

All statistical analyses were conducted using SAS Enterprise Guide (SAS Institute Inc., Cary, NC). A two‐tailed *p* value < 0.05 was considered statistically significant.

## 3 Results

### 3.1 Basic Characteristics of the Study Population

A total of 7411 patients with HCV infection were included in the final analysis, comprising 5785 patients who achieved SVR and 1676 who did not. The mean follow‐up period was 4.28 ± 2.79 years. The baseline demographic characteristics of the study population are summarized in Table 1.

**TABLE 1:** Patient demographics and baseline characteristics.

	Total (*n* = 7411)	SVR (*n* = 5785)	Non‐SVR (*n* = 1676)	*p*
Age, years	52.04 ± 11.42	51.50 ± 11.51	53.97 ± 10.91	< 0.0001
> 65	818 (11.0%)	605 (10.5%)	213 (13.1%)	0.003
Male	4173 (56.3%)	3320 (57.4%)	853 (52.5%)	0.0004
BMI (kg/m^2^)	24.65 ± 2.94	24.59 ± 2.90	24.89 ± 3.04	0.0001
≦ 18.5	110 (1.5%)	89 (1.5%)	21 (1.3%)	0.0004
> 18.5 and ≦ 24	2088 (28.2%)	1690 (29.2%)	398 (24.5%)	
> 24 and ≦ 27	4104 (55.4%)	3175 (54.9%)	929 (57.1%)	
> 27	1109 (15.0%)	831 (14.4%)	278 (17.1%)	
HCV genotype 1	3492 (50.3%)	2435 (45.0%)	1057 (68.8%)	< 0.0001
HCV RNA (log IU/mL)	5.74 ± 0.99	5.64 ± 1.01	6.08 ± 0.81	< 0.0001
AST (IU/L)	89.76 ± 62.44	90.13 ± 63.26	88.44 ± 59.44	0.3182
ALT (IU/L)	139.59 ± 109.38	144.04 ± 113.87	123.76 ± 89.87	< 0.0001
Platelet counts (×10^3^ U/L)	173.72 ± 52.75	175.64 ± 51.51	166.87 ± 56.44	< 0.0001
eGFR (mL/min/1.73 m^2^)	78.00 ± 24.45	78.06 ± 24.51	77.79 ± 24.22	0.698
< 60	1311 (17.7%)	1033 (17.9%)	278 (17.1%)	0.478
FIB‐4 score	2.76 ± 2.44	2.66 ± 2.40	3.12 ± 2.56	< 0.0001
> 3.25	1938 (26.2%)	1414 (24.4%)	524 (32.2%)	< 0.0001
Liver cirrhosis	962 (13.0%)	621 (10.7%)	341 (21.0%)	< 0.0001
Diabetes mellitus	648 (15.5%)	485 (14.9%)	163 (17.5%)	0.014
Hypertension	628 (15.0%)	485 (14.9%)	143 (15.4%)	0.742
Hyperlipidemia	316 (7.6%)	237 (7.3%)	79 (8.5%)	0.225
Statin user	356 (4.8%)	287 (5.0%)	69 (4.2%)	0.232
Aspirin user	582 (7.9%)	467 (8.1%)	115 (7.1%)	0.185
Follow‐up years (mean)	4.28 ± 2.79	4.36 ± 2.81	4.00 ± 2.68	< 0.0001
Follow‐up years (median, Q1–Q3)	4.00 (2.08–5.83)	4.08 (2.12–6.02)	3.73 (1.87–5.26)	
Follow‐up years (range)	16.96	16.96	13.68	

*Note*: Value expressed as mean ± SD or sample number and proportions.

Abbreviations: ALT, aspartate aminotransferase; AST, alanine aminotransferase; BMI, body mass index; eGFR, estimated glomerular filtration rate; FIB‐4, fibrosis‐4; HCV, hepatic C virus; SVR, sustained virological response.

The mean age was 52.04 years, and 56.3% of participants were male. The mean BMI was 24.65 ± 2.94 kg/m^2^. The mean baseline HCV viral load was 5.74 log IU/mL, and 50.3% of patients were infected with HCV genotype 1. The mean FIB‐4 was 2.76 ± 2.44, and 13.0% of patients had liver cirrhosis.

Comparisons between patients with and without SVR are also presented in Table 1. Factors associated with non‐SVR included older age, female sex, higher BMI, HCV genotype 1, higher viral load, lower alanine aminotransferase (ALT) levels, lower platelet counts, and more advanced fibrosis or cirrhosis.

### 3.2 Cumulative Incidence of Cardiovascular Disease Events After Interferon‐Based Therapy and the Effects of Achieving an SVR

Among the 7411 included patients, 5785 (78.1%) achieved SVR following IFN‐based therapy. During a mean follow‐up period of 4.28 years, 647 patients (11.2%) in the SVR group and 166 patients (10.2%) in the non‐SVR group developed new‐onset CVD (Figure 1). The annual incidence rates were 256.4 and 255.4 per 10,000 person‐years, respectively (Table 1).

**FIGURE 2:**
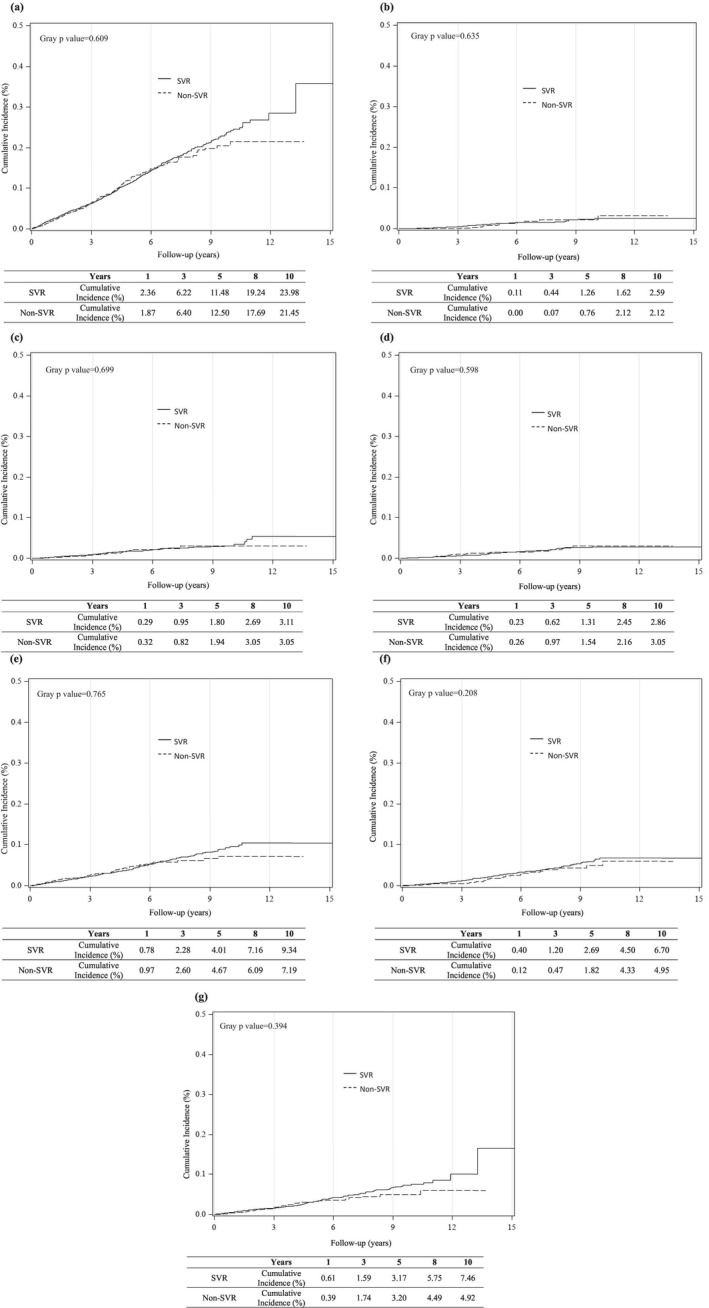
Cumulative incidence of new‐onset cardiovascular disease (CVD) between sustained virologic response (SVR) and non‐SVR patients after chronic hepatitis C (CHC) patients who achieved antiviral therapy, considering death as a competing risk. (a) All CVDs, (b) stroke, (c) peripheral arterial occlusive disease, (d) heart failure, (e) arrhythmia, (f) cerebrovascular accident, (g) coronary artery disease.

After adjusting for death as a competing risk, the cumulative incidence rates of CVD at 1, 3, 5, 8, and 10 years among SVR patients were 2.36%, 6.22%, 11.48%, 19.24%, and 23.98%, respectively, which were comparable to 1.87%, 6.40%, 12.50%, 17.69%, and 21.45% in the non‐SVR group (*p* value for Gray's method = 0.609, Figure 2a). There was no statistically significant difference in the cumulative incidence of CVD between the SVR and non‐SVR groups.

Further analysis stratified by CVD subtypes (stroke, PAOD, heart failure, arrhythmia, cerebrovascular accident, and coronary artery disease) revealed that IFN‐based therapy and attainment of SVR were not significantly associated with CVD event rates across any subtype (Figure 2b–g, Table 2).

**TABLE 2:** The effects of sustained virologic response for new‐onset cardiovascular disease (death as competing risk).

*N* (% per 10,000 person‐years)	Total (*N* = 7411)	SVR (*N* = 5785)	Non‐SVR (*N* = 1626)	Crude HR (95% CI)	*p*
All CVDs	813 (256.2)	647 (256.4)	166 (255.4)	0.96 (0.81–1.14)	0.605
Stroke	72 (21.9)	61 (22.3)	14 (21.0)	0.87 (0.49–1.54)	0.632
PAOD	122 (35.9)	98 (36.2)	24 (31.9)	0.92 (0.59–1.43)	0.699
Heart failure	95 (27.9)	73 (26.9)	22 (31.7)	1.14 (0.71–1.82)	0.602
Arrhythmia	299 (89.5)	238 (89.5)	61 (89.4)	0.96 (0.72–1.27)	0.762
Cerebrovascular disease	181 (53.4)	150 (55.6)	31 (44.9)	0.78 (0.53–1.15)	0.207
Coronary artery disease	230 (68.2)	187 (69.7)	43 (62.6)	0.86 (0.62–1.20)	0.387

Abbreviations: CI, confidence intervals; CVD, cardiovascular disease; HR, hazard ratio; PAOD, peripheral arterial occlusive disease; SVR, sustained virological response.

Subgroup analyses were conducted by stratifying patients according to gender, age (< 65 vs. ≥ 65 years), BMI (≤ 24, 24–27, > 27 kg/m^2^), HCV genotype (1 vs. non‐1), eGFR (< 60 vs. ≥ 60 mL/min/1.73m^2^), and FIB‐4 (< 3.25 vs. ≥ 3.25). In most subgroups, achievement of SVR did not significantly impact the risk of new‐onset CVD.

However, in certain subgroups, IFN‐based therapy and attainment of SVR were unexpectedly associated with a higher CVD event rate compared to the non‐SVR group. In patients with FIB‐4 ≥ 3.25, those who achieved SVR had a higher incidence of CVD events (HR = 1.45, CI = 1.06–1.96, *p* = 0.017). In male patients, SVR was associated with an increased incidence of PAOD (HR = 2.63, CI = 1.04–6.67, *p* = 0.041) (Table S2a–g).

### 3.3 Effect of SVR on CVD Events Using Proportional Hazards Model According to Covariate Adjustment

Three Cox proportional hazards models were constructed to assess the association between SVR and the incidence of CVD events, adjusting for traditional and additional CVD risk factors.

In Model 1, which adjusted for age, sex, BMI, statin use, and aspirin use, treatment with IFN‐based therapy was associated with an adjusted HR of 0.88 (95% CI: 0.71–1.05; *p*: 0.158) for all CVD events, 0.88 (95% CI: 0.50–1.56; *p*: 0.670) for stroke, 0.82 (95% CI: 0.52–1.82; *p*: 0.380) for PAOD, 1.03 (95% CI: 0.63–1.67; *p*: 0.911) for heart failure, 0.91 (95% CI: 0.68–1.20; *p*: 0.486) for arrhythmia, 0.75 (95% CI: 0.51–1.10; *p*: 0.135) for cerebrovascular disease, 0.87 (95% CI: 0.62–1.20; *p*: 0.407) for coronary artery disease (Table 3). There was no statistically significant association between attainment of SVR and the incidence rates of any CVD subtypes (Table 3).

**TABLE 3:** Effect of SVR on CVD events using the proportional hazards model accounting for competing death risk, according to covariate adjustment.

	Model 1 (*n* = 7411)		Model 2 (*n* = 6946)		Model 3 (*n* = 4018)	
	Adjusted HR (95% CI)	Adjusted *p*	Adjusted HR (95% CI)	Adjusted *p*	Adjusted HR (95% CI)	Adjusted *p*
All CVDs	0.88 (0.74–1.05)	0.158	0.92 (0.71–1.11)	0.402	0.86 (0.67–1.10)	0.217
Stroke	0.88 (0.50–1.56)	0.670	0.63 (0.31–1.25)	0.184	0.50 (0.13–1.56)	0.214
PAOD	0.82 (0.52–1.82)	0.380	0.83 (0.51–1.37)	0.469	0.93 (0.47–1.82)	0.835
Heart failure	1.03 (0.63–1.67)	0.911	1.14 (0.69–1.89)	0.604	1.27 (0.69–2.33)	0.451
Arrhythmia	0.91 (0.68–1.20)	0.486	0.94 (0.70–1.27)	0.659	0.90 (0.62–1.41)	0.592
Cerebrovascular disease	0.75 (0.51–1.10)	0.135	0.72 (0.47–1.10)	0.125	0.55 (0.28–1.06)	0.076
Coronary artery disease	0.87 (0.62–1.20)	0.407	0.91 (0.64–1.30)	0.605	0.81 (0.50–1.82)	0.374

*Note*: Model 1 was adjusted for age, sex, BMI, statin use (yes/no), aspirin use (yes/no). Model 2 was adjusted for all covariates included in Model 1, and FIB‐4, eGFR, HCV genotype (1 vs. non‐1). Model 3 was adjusted for all covariates included in Model 2, and diabetes mellitus (yes/no), hypertension (yes/no), and hyperlipidemia (yes/no).

Abbreviations: BMI, body mass index; CI, confidence intervals; CVD, cardiovascular disease; eGFR, estimated glomerular filtration rate; FIB‐4, fibrosis‐4; HCV, hepatitis C virus; HR, hazard ratio; PAOD, peripheral arterial occlusive disease; SVR, sustained virological response.

Additional CVD risk factors were incorporated as confounding variables in Model 2 (adjusted for FIB‐4, eGFR, and HCV genotype) and Model 3 (further adjusted for diabetes mellitus, hypertension, and hyperlipidemia). The results remained consistent across all models, with no statistically significant association between SVR and the risk of CVD events. Although Model 3 exhibited a lower HR for cerebrovascular disease compared to Model 2 and Model 1, the association did not reach statistical significance (HR = 0.55, 95% CI: 0.28–1.06, *p* = 0.076, Table 2).

### 3.4 Sensitivity Analysis

To account for potential adverse effects following IFN‐based therapy, a sensitivity analysis was conducted by excluding CVD events that occurred within the first year after antiviral therapy.

The results remained consistent with the primary analysis. There was no statistically significant difference in the incidence of CVD events between the SVR and non‐SVR groups across all individual CVD subtypes (Table 4). Furthermore, in the three Cox proportional hazards models adjusted for CVD risk factors, no significant association was observed between SVR and a reduced risk of CVD events (Table 5). The findings remained robust after sensitivity analysis.

**TABLE 4:** The effects of sustained virologic response for new‐onset CVDs by sensitivity analysis (excluded the events occur within 1 year post antiviral therapy).

*N* (% per 10,000 person‐years)	Total (*N* = 7237)	SVR (*N* = 5643)	Non‐SVR (*N* = 1594)	Crude HR (95% CI)	*p*
All CVDs	650	514	136	1.01 (0.84–1.23)	0.896
Stroke	67	53	14	1.01 (0.56–1.82)	0.962
PAOD	97	78	19	0.93 (0.56–1.54)	0.763
Heart failure	74	57	17	1.15 (0.67–1.96)	0.611
Arrhythmia	235	189	46	0.93 (0.67–1.28)	0.649
Cerebrovascular disease	152	124	28	0.87 (0.58–1.32)	0.503
Coronary artery disease	182	147	35	0.91 (0.63–1.32)	0.634

Abbreviations: CI, confidence intervals; CVD, cardiovascular disease; HR, hazard ratio; PAOD, peripheral arterial occlusive disease; SVR, sustained virological response.

**TABLE 5:** Effect of SVR on CVD events using proportional hazards model and according to covariate adjustment, by sensitivity analysis (excluded the events occur within 1 year post antiviral therapy).

	Model 1 (*n* = 7237)		Model 2 (*n* = 6616)		Model 3 (*n* = 3104)	
	Adjusted HR (95% CI)	Adjusted *p*	Adjusted HR (95% CI)	Adjusted *p*	Adjusted HR (95% CI)	Adjusted *p*
All CVDs	0.93 (0.77–1.14)	0.476	0.96 (0.78–1.18)	0.671	0.88 (0.67–1.15)	0.354
Stroke	1.00 (0.56–1.79)	0.9995	0.74 (0.36–1.52)	0.413	0.53 (0.16–1.79)	0.307
PAOD	0.82 (0.50–1.37)	0.451	0.86 (0.49–1.47)	0.577	0.97 (0.46–2.04)	0.943
Heart failure	1.04 (0.60–1.79)	0.898	1.06 (0.59–1.89)	0.844	1.23 (0.60–2.05)	0.576
Arrhythmia	0.88 (0.63–1.20)	0.429	0.88 (0.62–1.23)	0.445	0.84 (0.54–1.28)	0.413
Cerebrovascular disease	0.81 (0.54–1.22)	0.322	0.77 (0.49–1.20)	0.253	0.59 (0.29–1.20)	0.144
Coronary artery disease	0.89 (0.61–1.30)	0.543	0.91 (0.61–1.35)	0.652	0.76 (0.44–1.30)	0.315

*Note*: Model 1 was adjusted for age, sex, BMI, statin use (yes/no), aspirin use (yes/no). Model 2 was adjusted for all covariates included in Model 1, and FIB‐4, eGFR, HCV genotype (1 vs. non‐1). Model 3 was adjusted for all covariates included in Model 2, and diabetes mellitus (yes/no), hypertension (yes/no), and hyperlipidemia (yes/no).

Abbreviations: BMI, body mass index; CI, confidence intervals; CVD, cardiovascular disease; eGFR, estimated glomerular filtration rate; FIB‐4, fibrosis‐4; HCV, hepatitis C virus; HR, hazard ratio; PAOD, peripheral arterial occlusive disease; SVR, sustained virological response.

## 4 Discussion

Our cohort study yielded several key results. First, patients with HCV who received IFN‐based therapy did not show a significant reduction in CVD risk. Three Cox proportional hazards models adjusted for CVD risk factors were used to assess the overall and subtype‐specific CVD risks, and the results remained consistent across all models (Table 3). Second, patients who achieved SVR exhibited a lower risk of stroke than those who did not; this trend persisted even after adjusting for CVD risk factors, although the difference was not statistically significant (Table 3). Finally, to mitigate potential bias and account for the adverse effects of IFN‐based therapy, we conducted a sensitivity analysis excluding new‐onset CVD events within one year post‐treatment (Tables 4 and 5). These findings remained unchanged, reinforcing the robustness of our results.

CVD remains a significant global health challenge, with a high prevalence among HCV patients [25]. Studies have suggested that HCV eradication reduces CVD risk, particularly when SVR is achieved through antiviral therapy [26]. Previous analyses further confirmed this association, demonstrating that SVR is associated with a lower incidence of CVD, coronary artery disease, and stroke [27]. However, these studies did not account for the potential variations in the cardiovascular effects of different antiviral agents, which may have influenced the observed outcomes. The effect of DAA versus IFN‐based therapies on cardiovascular risk reduction remains a critical consideration when interpreting these findings. A large American cohort study (*n* > 16,000) reported a 50% reduction in CVD events among patients diagnosed with HCV (7.2% vs. 13.8%, *p* < 0.0001), with DAA therapy demonstrating greater cardiovascular benefits than IFN‐based therapy [26].

Our findings differ from those of previous studies as we specifically examined the impact of IFN‐based therapy on CVD risk in patients with HCV. Moreover, we utilized comprehensive viral load data to assess SVR accurately. We found that even among those who achieved SVR, the CVD risk reduction was insignificant (Figure 2).

While a meta‐analysis suggested that HCV treatment lowers the CVD risk, many of the included studies lacked SVR stratification, included co‐infected populations, or used heterogeneous treatment regimens [27]. In contrast, our study precisely defined virological characteristics, incorporated accurate viral load measurements, and focused exclusively on IFN‐treated HCV patients, minimizing confounding factors and enhancing the accuracy of the results.

Research on the effect of IFN‐based therapy on cardiovascular outcomes in patients with HCV has yielded inconsistent results. While HCV eradication is generally considered to reduce CVD risk [28, 29], studies focusing on IFN‐treated populations have not demonstrated a definitive reduction in cardiovascular events. Mahale et al. [30] reported that although SVR reduced stroke risk (HR = 0.84, 95% CI: 0.74–0.94), it did not significantly lower overall CVD risk (HR = 1.12, 95% CI: 0.81–1.56). Similarly, Leone et al. [14] found no significant reduction in cardiovascular risk between the SVR and non‐SVR groups (HR = 1.14, 95% CI: 0.57–2.3). The uncertainty in findings may be attributed to IFN‐α‐induced vascular damage, as IFN‐α has been linked to endothelial dysfunction, particularly impairing endothelial progenitor cells and circulating angiogenic cells, both essential for vascular repair. IFN therapy is also associated with cardiac arrhythmia, impaired cardiac function, and myocardial ischemia [31]. A Japanese cohort study identified an association between IFN therapy and CVD events in 3.2% of the patients with HCV infection. Although symptoms may improve after IFN discontinuation, cardiovascular toxicity may persist after post‐treatment [17]. These findings suggest that IFN‐based therapy does not consistently reduce the CVD risk and may contribute to additional CV complications, warranting further investigation.

In Taiwan, Hsu C.‐S. et al. [32] conducted a large real‐world cohort study (*n* = 3113) using data from the Taiwan National Health Insurance Program and reported that IFN treatment was associated with a 61% reduction in stroke risk in HCV‐infected patients. However, patient classification was based on treatment status rather than SVR. Similarly, another large Taiwanese cohort study linked HCV treatment with a lower incidence of acute coronary syndrome, heart failure, venous thromboembolism, stroke, and cardiovascular death. However, patient categorization remains focused on treatment versus no treatment without accounting for SVR status [33].

To our knowledge, this study is one of the largest multicenter investigations to assess the long‐term SVR outcomes of new‐onset CVD events in patients with HCV. To minimize data bias, we used three Cox proportional hazards models with univariate and multivariate adjustments for all covariates. Despite these refinements, our findings remained robust and reproducible across all models (Table 3), thus strengthening the reliability of our conclusions.

This study has several strengths. First, this was a large multicenter study evaluating the impact of SVR in patients with HCV receiving IFN‐based therapy for CVD. In Taiwan, the T‐COACH serves as the largest nationwide HCV registry cohort, offering comprehensive patient characteristics and treatment outcomes. Second, the SVR status was directly assessed using viral load measurements rather than treatment status, ensuring a more precise evaluation of the virologic response. Third, we conducted a sensitivity analysis and constructed three Cox proportional hazards models to minimize data bias related to the impact of SVR and IFN‐associated adverse effects (Tables 4 and 5). We analyzed various CVD events individually, allowing for a detailed assessment of their association with SVR (Figure 2). These analyses enhance our understanding of the complex relationship between SVR and CVD risk in IFN‐treated HCV patients.

This study had certain limitations. First, CVD events were identified using the ICD‐9‐CM or drug codes, which may introduce variability in cardiovascular diagnoses across healthcare systems. However, all diagnoses were confirmed based on at least three outpatient visits or one hospitalization, ensuring a reasonable level of diagnostic reliability. Second, the IFN treatment itself may have influenced the risk of CVD, potentially confounding our findings. To address this, we performed a sensitivity analysis, excluding CVD events occurring within 1 year post‐treatment (Tables 4 and 5), to minimize potential bias. Finally, the relatively short follow‐up period limited the ability to assess long‐term cardiovascular outcomes. Further large‐scale studies with extended follow‐up periods are required to confirm these findings.

## 5 Conclusions

In this large, nationwide cohort study, achieving SVR following IFN‐based therapy did not significantly reduce CVD risk in patients with HCV. The cumulative incidence of CVD events remained comparable between the SVR and non‐SVR groups even after adjusting for confounders. However, a lower risk of stroke was observed in the patients who achieved SVR, although this trend was not statistically significant. These findings emphasize the need for further investigation of the long‐term cardiovascular effects of HCV eradication, particularly in the era of DAA, which may offer greater cardiovascular protection than IFN‐based therapies.

## Conflicts of Interest

The authors declare no conflicts of interest.

## Supporting information


**Data S1.** kjm270036‐sup‐0001‐Tables.

## Data Availability

The data that support the findings of this study are available on request from the corresponding author. The data are not publicly available due to privacy or ethical restrictions.
