# Supplementary material for: Impact of Hepatitis C Virus Clearance on Cardiovascular Risk: A Real‐World Experience From the Nationwide Taiwan Hepatitis C Virus Registry
Source: Kaohsiung J Med Sci. 2025 May 24;41(8):e70036. doi: 10.1002/kjm2.70036 (PMC12407379; doi:10.1002/kjm2.70036)
Supplement: Supplementary file 1 — Data S1. kjm270036‐sup‐0001‐Tables. [file KJM2-41-e70036-s001.doc]

**Supple Table 1. The diagnosis of CVD events according to the ICD-9 codes**

| Events | ICD-9 codes |
| --- | --- |
| All CVDs | 410-414, 426-428, 430-438, 440.2, 440.3, 440.8, 443.81, 443.89, 443.9, 444.22, 444.8, 447.8, 447.9 |
| Stroke | 433, 434 |
| PAOD | 440.2, 440.3, 440.8, 443.81, 443.89, 443.9, 444.22, 444.8, 447.8, 447.9 |
| Heart failure | 428 |
| Arrhythmia | 426-427 |
| Cerebrovascular disease | 430-438 |
| Coronary artery disease | 410-413 |
| Abbreviation: CVD, cardiovascular disease; PAOD, peripheral arterial occlusive disease | |

**Supplement Table 2. The effects of sustained virologic response for new-onset cardiovascular disease (CVD) among subgroups. (a) all CVDs. (b) stroke. (c) peripheral arterial occlusive disease. (d) heart failure. (e) arrhythmia. (f) cerebrovascular accident. (g) coronary artery disease.**

Abbreviation: SVR, sustained virological response; HR, hazard ratio; CI, confidence intervals; BMI, body mass index; HCV, hepatitis C virus; eGFR, estimated glomerular filtration rate; FIB-4, fibrosis-4.

1. **all CVDs**

|  |
| --- |

|  |  | Events, % | | Crude ( SVR vs non-SVR) | |
| --- | --- | --- | --- | --- | --- |
|  |  | SVR | Non-SVR | HR (95% CI) | P value |
| Gender |  |  |  |  |  |
|  | Male | 10.3 | 8.3 | 1.20 (0.93-1.56) | 0.156 |
|  | Female | 12.4 | 12.3 | 0.94 (0.75-1.19) | 0.603 |
| Age |  |  |  |  |  |
|  | ≦ 65 | 10.3 | 9.6 | 1.01 (0.83-1.22) | 0.942 |
|  | > 65 | 18.5 | 14.6 | 1.47 (0.99-2.17) | 0.054 |
| BMI |  |  |  |  |  |
|  | ≦ 24 | 10.3 | 10.7 | 0.81 (0.58-1.12) | 0.215 |
|  | 24-27 | 10.5 | 10.2 | 1.06 (0.85-1.33) | 0.574 |
|  | > 27 | 15.6 | 9.4 | 1.49 (0.97-2.27) | 0.065 |
| HCV genotype |  |  |  |  |  |
|  | 1 | 9.8 | 9.7 | 1.03 (0.77-1.37) | 0.853 |
|  | Non-1 | 12.2 | 11.1 | 1.15 (0.60-2.22) | 0.666 |
| eGFR |  |  |  |  |  |
|  | >60 | 10.7 | 10.2 | 0.99 (0.82-1.19) | 0.906 |
|  | ≦ 60 | 13.7 | 10.4 | 1.32 (0.88-1.96) | 0.187 |
| FIB-4 |  |  |  |  |  |
|  | <3.25 | 10.2 | 10.3 | 0.91 (0.74-1.12) | 0.376 |
|  | >=3.25 | 14.3 | 10.1 | 1.45 (1.06-1.96) | 0.017 |

**(b) stroke**

|  |  | Events, % | | Crude (SVR vs non-SVR) | |
| --- | --- | --- | --- | --- | --- |
|  |  | SVR | Non-SVR | HR (95% CI) | P value |
| Gender |  |  |  |  |  |
|  | Male | 1.4 | 1.2 | 1.15 (0.57-2.22) | 0.729 |
|  | Female | 0.6 | 0.5 | 1.09 (0.36-3.23) | 0.880 |
| Age |  |  |  |  |  |
|  | ≦ 65 | 0.8 | 0.6 | 1.35 (0.63-2.86) | 0.440 |
|  | > 65 | 3.0 | 2.8 | 1.15 (0.47-2.86) | 0.754 |
| BMI |  |  |  |  |  |
|  | ≦ 24 | 1.0 | 0.7 | 1.08 (0.31-3.57) | 0.909 |
|  | 24-27 | 1.1 | 1.1 | 1.09 (0.54-2.17) | 0.804 |
|  | > 27 | 1.0 | 0.4 | 2.27 (0.28-16.67) | 0.439 |
| HCV genotype |  |  |  |  |  |
|  | 1 | 1.1 | 0.6 | 1.79 (0.74-4.35) | 0.199 |
|  | Non-1 | 0.9 | 0.8 | 1.02 (0.36-2.86) | 0.973 |
| eGFR |  |  |  |  |  |
|  | >60 | 0.9 | 0.45 | 1.85 (0.78-4.35) | 0.166 |
|  | ≦ 60 | 1.9 | 2.9 | 0.63 (0.28-1.43) | 0.266 |
| FIB-4 |  |  |  |  |  |
|  | <3.25 | 0.8 | 0.9 | 0.81 (0.40-1.67) | 0.573 |
|  | >=3.25 | 1.8 | 0.8 | 2.33 (0.82-6.67) | 0.110 |

**(c) peripheral arterial occlusive disease**

|  |  | Events, % | | Crude( SVR vs non-SVR) | |
| --- | --- | --- | --- | --- | --- |
|  |  | SVR | Non-SVR | HR (95% CI) | P value |
| Gender |  |  |  |  |  |
|  | Male | 1.6 | 0.6 | 2.63 (1.04-6.67) | 0.041 |
|  | Female | 1.9 | 2.5 | 0.69 (0.41-1.19) | 0.184 |
| Age |  |  |  |  |  |
|  | ≦ 65 | 1.5 | 1.3 | 1.08 (0.22-1.82) | 0.766 |
|  | > 65 | 3.6 | 2.8 | 1.41 (0.56-3.57) | 0.470 |
| BMI |  |  |  |  |  |
|  | ≦ 24 | 1.4 | 1.9 | 0.63 (0.28-1.39) | 0.248 |
|  | 24-27 | 2.0 | 1.4 | 1.45 (0.79-2.63) | 0.227 |
|  | > 27 | 1.3 | 1.1 | 1.02 (0.28-3.70) | 0.979 |
| HCV genotype |  |  |  |  |  |
|  | 1 | 1.4 | 1.1 | 1.22 (0.63-2.32) | 0.551 |
|  | Non-1 | 2.0 | 1.9 | 0.95 (0.47-1.92) | 0.886 |
| eGFR |  |  |  |  |  |
|  | >60 | 1.7 | 1.6 | 1.00 (0.62-1.61) | 0.992 |
|  | ≦ 60 | 1.7 | 0.7 | 2.17 (0.5-9.09) | 0.300 |
| FIB-4 |  |  |  |  |  |
|  | <3.25 | 1.4 | 1.2 | 1.11 (0.61-2.04) | 0.719 |
|  | >=3.25 | 2.6 | 2.1 | 1.20 (0.61-2.38) | 0.598 |

**(d) heart failure**

|  |  | Events, % | | Crude (SVR vs non-SVR) | |
| --- | --- | --- | --- | --- | --- |
|  |  | SVR | Non-SVR | HR (95% CI) | P value |
| Gender |  |  |  |  |  |
|  | Male | 1.0 | 1.5 | 0.64 (0.34-1.22) | 0.174 |
|  | Female | 1.6 | 1.2 | 1.28 (0.62-2.63) | 0.505 |
| Age |  |  |  |  |  |
|  | ≦ 65 | 1.2 | 1.3 | 0.85 (0.51-1.45) | 0.562 |
|  | > 65 | 2.0 | 1.9 | 1.12 (0.37-3.45) | 0.833 |
| BMI |  |  |  |  |  |
|  | ≦ 24 | 1.0 | 1.7 | 0.51 (0.22-1.23) | 0.134 |
|  | 24-27 | 1.0 | 1.2 | 0.91 (0.46-1.67) | 0.777 |
|  | > 27 | 2.7 | 1.4 | 1.52 (0.53-4.35) | 0.435 |
| HCV genotype |  |  |  |  |  |
|  | 1 | 0.7 | 1.3 | 1.92 (0.96-3.84) | 0.066 |
|  | Non-1 | 1.6 | 1.3 | 0.52 (0.26-1.04) | 0.770 |
| eGFR |  |  |  |  |  |
|  | >60 | 1.1 | 1.1 | 0.96 (0.54-1.69) | 0.894 |
|  | ≦ 60 | 1.8 | 2.5 | 0.70 (0.30-1.67) | 0.425 |
| FIB-4 |  |  |  |  |  |
|  | <3.25 | 1.0 | 1.2 | 0.81 (0.44-1.49) | 0.508 |
|  | >=3.25 | 2.0 | 1.7 | 1.11 (0.53-2.33) | 0.778 |

**(e) arrhythmia**

|  |  | Events, % | | Crude (SVR vs non-SVR) | |
| --- | --- | --- | --- | --- | --- |
|  |  | SVR | Non-SVR | HR (95% CI) | P value |
| Gender |  |  |  |  |  |
|  | Male | 3.8 | 2.7 | 1.37 (0.88-2.13) | 0.159 |
|  | Female | 4.5 | 4.9 | 0.85 (0.59-1.23) | 0.407 |
| Age |  |  |  |  |  |
|  | ≦ 65 | 4.0 | 3.3 | 1.15 (0.83-1.59) | 0.388 |
|  | > 65 | 5.1 | 7.0 | 0.78 (0.42-1.43) | 0.420 |
| BMI |  |  |  |  |  |
|  | ≦ 24 | 4.3 | 4.5 | 0.82 (0.50-1.35) | 0.443 |
|  | 24-27 | 3.6 | 3.2 | 1.15 (0.77-1.72) | 0.492 |
|  | > 27 | 5.7 | 4.3 | 1.11 (0.59-2.08) | 0.752 |
| HCV genotype |  |  |  |  |  |
|  | 1 | 3.6 | 4.0 | 0.86 (0.60-1.25) | 0.438 |
|  | Non-1 | 4.6 | 3.3 | 1.27 (0.75-2.13) | 0.379 |
| eGFR |  |  |  |  |  |
|  | >60 | 4.0 | 3.6 | 1.05 (0.76-1.43) | 0.769 |
|  | ≦ 60 | 4.6 | 4.3 | 1.02 (0.54-1.92) | 0.949 |
| FIB-4 |  |  |  |  |  |
|  | <3.25 | 3.6 | 3.5 | 0.96 (0.68-1.37) | 0.844 |
|  | >=3.25 | 5.7 | 4.4 | 1.30 (0.81-2.04) | 0.278 |

**(f) cerebrovascular accident**

|  |  | Events, % | | Crude (SVR vs non-SVR) | |
| --- | --- | --- | --- | --- | --- |
|  |  | SVR | Non-SVR | HR (95% CI) | P value |
| Gender |  |  |  |  |  |
|  | Male | 2.6 | 1.9 | 1.35 (0.79-2.33) | 0.269 |
|  | Female | 2.5 | 1.9 | 1.20 (0.69-2.13) | 0.513 |
| Age |  |  |  |  |  |
|  | ≦ 65 | 2.2 | 1.5 | 1.35 (0.85-2.17) | 0.206 |
|  | > 65 | 6.1 | 4.7 | 1.45 (0.74-2.86) | 0.287 |
| BMI |  |  |  |  |  |
|  | ≦ 24 | 2.5 | 1.7 | 1.25 (0.56-2.78) | 0.579 |
|  | 24-27 | 2.6 | 2.4 | 1.12 (0.7-2.0) | 0.637 |
|  | > 27 | 2.9 | 0.7 | 3.45 (0.81-14.29) | 0.093 |
| HCV genotype |  |  |  |  |  |
|  | 1 | 2.2 | 1.6 | 1.30 (0.76-2.27) | 0.336 |
|  | Non-1 | 2.8 | 2.1 | 1.20 (0.63-2.33) | 0.573 |
| eGFR |  |  |  |  |  |
|  | >60 | 2.3 | 1.5 | 1.45 (0.90-2.33) | 0.122 |
|  | ≦ 60 | 4.0 | 4.0 | 0.95 (0.49-1.85) | 0.881 |
| FIB-4 |  |  |  |  |  |
|  | <3.25 | 2.1 | 1.7 | 1.12 (0.69-1.85) | 0.627 |
|  | >=3.25 | 4.0 | 2.3 | 1.72 (0.93-3.23) | 0.082 |

**(g) coronary artery disease.**

|  |  | Events, % | | Crude (SVR vs non-SVR) | |
| --- | --- | --- | --- | --- | --- |
|  |  | SVR | Non-SVR | HR (95% CI) | P value |
| Gender |  |  |  |  |  |
|  | Male | 3.5 | 2.2 | 1.52 (0.93-2.44) | 0.092 |
|  | Female | 2.8 | 3.1 | 0.85 (0.53-1.35) | 0.482 |
| Age |  |  |  |  |  |
|  | ≦ 65 | 3.1 | 2.7 | 1.06 (0.75-1.52) | 0.726 |
|  | > 65 | 4.5 | 3.4 | 2.04 (0.79-5.26) | 0.139 |
| BMI |  |  |  |  |  |
|  | ≦ 24 | 3.0 | 2.6 | 0.98 (0.51-1.89) | 0.953 |
|  | 24-27 | 3.0 | 2.4 | 1.33 (0.83-2.13) | 0.228 |
|  | > 27 | 4.5 | 3.6 | 1.10 (0.54-2.22) | 0.798 |
| HCV genotype |  |  |  |  |  |
|  | 1 | 2.9 | 2.5 | 1.12 (0.72-1.75) | 0.606 |
|  | Non-1 | 3.5 | 3.1 | 1.03 (0.60-1.75) | 0.918 |
| eGFR |  |  |  |  |  |
|  | >60 | 3.0 | 2.8 | 0.99 (0.69-1.43) | 0.979 |
|  | ≦ 60 | 4.4 | 1.8 | 2.38 (0.93-5.88) | 0.068 |
| FIB-4 |  |  |  |  |  |
|  | <3.25 | 3.2 | 3.1 | 0.93 (0.65-1.37) | 0.732 |
|  | >=3.25 | 3.5 | 1.7 | 2.0 (0.98-4.00) | 0.056 |
